# Persistent endotheliopathy in the pathogenesis of long COVID syndrome

**DOI:** 10.1111/jth.15490

**Published:** 2021-09-12

**Authors:** Helen Fogarty, Liam Townsend, Hannah Morrin, Azaz Ahmad, Claire Comerford, Ellie Karampini, Hanna Englert, Mary Byrne, Colm Bergin, Jamie M. O’Sullivan, Ignacio Martin‐Loeches, Parthiban Nadarajan, Ciaran Bannan, Patrick W. Mallon, Gerard F. Curley, Roger J. S. Preston, Aisling M. Rehill, Dennis McGonagle, Cliona Ni Cheallaigh, Ross I. Baker, Thomas Renné, Soracha E. Ward, James S. O’Donnell, the Irish COVID‐19 Vasculopathy Study (iCVS) investigators, Niamh O’Connell, Kevin Ryan, Dermot Kenny, Judicael Fazavana

**Affiliations:** ^1^ Irish Centre for Vascular Biology School of Pharmacy and Biomolecular Sciences Royal College of Surgeons in Ireland Dublin Ireland; ^2^ Department of Infectious Diseases St James’s Hospital Dublin Ireland; ^3^ Department of Clinical Medicine School of Medicine Trinity Translational Medicine Institute Trinity College Dublin Dublin Ireland; ^4^ Institute of Clinical Chemistry and Laboratory Medicine University Medical Center Hamburg‐ Eppendorf Hamburg Germany; ^5^ National Coagulation Centre St James’s Hospital Dublin Ireland; ^6^ Department of Intensive Care Medicine St James’s Hospital Dublin Ireland; ^7^ Department of Respiratory Medicine St James’s Hospital Dublin Ireland; ^8^ Centre for Experimental Pathogen Host Research University College Dublin Dublin Ireland; ^9^ St Vincent's University Hospital Dublin Ireland; ^10^ Department of Anaesthesia and Critical Care Royal College of Surgeons in Ireland Dublin Ireland; ^11^ National Children’s Research Centre Our Lady’s Children’s Hospital Crumlin Dublin Ireland; ^12^ Leeds Institute of Rheumatic and Musculoskeletal Medicine (LIRMM) University of Leeds Leeds UK; ^13^ National Institute for Health Research (NIHR) Leeds Biomedical Research Centre (BRC) Leeds Teaching Hospitals Leeds UK; ^14^ Western Australia Centre for Thrombosis and Haemostasis Perth Blood Institute Murdoch University Perth WA Australia; ^15^ Irish‐Australian Blood Collaborative (IABC) Network Dublin Ireland; ^16^ Center for Thrombosis and Hemostasis (CTH) Johannes Gutenberg University Medical Center Mainz Germany

**Keywords:** convalescent COVID‐19, endothelial cell (EC) activation, long COVID

## Abstract

**Background:**

Persistent symptoms including breathlessness, fatigue, and decreased exercise tolerance have been reported in patients after acute SARS‐CoV‐2 infection. The biological mechanisms underlying this “long COVID” syndrome remain unknown. However, autopsy studies have highlighted the key roles played by pulmonary endotheliopathy and microvascular immunothrombosis in acute COVID‐19.

**Objectives:**

To assess whether endothelial cell activation may be sustained in convalescent COVID‐19 patients and contribute to long COVID pathogenesis.

**Patients and Methods:**

Fifty patients were reviewed at a median of 68 days following SARS‐CoV‐2 infection. In addition to clinical workup, acute phase markers, endothelial cell (EC) activation and NETosis parameters and thrombin generation were assessed.

**Results:**

Thrombin generation assays revealed significantly shorter lag times (*p* < .0001, 95% CI −2.57 to −1.02 min), increased endogenous thrombin potential (*p* = .04, 95% CI 15–416 nM/min), and peak thrombin (*p* < .0001, 95% CI 39–93 nM) in convalescent COVID‐19 patients. These prothrombotic changes were independent of ongoing acute phase response or active NETosis. Importantly, EC biomarkers including von Willebrand factor antigen (VWF:Ag), VWF propeptide (VWFpp), and factor VIII were significantly elevated in convalescent COVID‐19 compared with controls (*p* = .004, 95% CI 0.09–0.57 IU/ml; *p* = .009, 95% CI 0.06–0.5 IU/ml; *p* = .04, 95% CI 0.03–0.44 IU/ml, respectively). In addition, plasma soluble thrombomodulin levels were significantly elevated in convalescent COVID‐19 (*p* = .02, 95% CI 0.01–2.7 ng/ml). Sustained endotheliopathy was more frequent in older, comorbid patients, and those requiring hospitalization. Finally, both plasma VWF:Ag and VWFpp levels correlated inversely with 6‐min walk tests.

**Conclusions:**

Collectively, our findings demonstrate that sustained endotheliopathy is common in convalescent COVID‐19 and raise the intriguing possibility that this may contribute to long COVID pathogenesis.

**ESSENTIALS**

- Ongoing endotheliopathy is a common finding in convalescent COVID‐19 patients and is independent of the acute phase response.
- Plasma FVIII:C levels and thrombin generation are significantly increased in convalescent COVID‐19 patients compared to healthy controls.
- Plasma VWF:Ag, VWFpp and sTM levels remain persistently elevated in a proportion of patients following apparent resolution of acute COVID‐19.
- Markers of endotheliopathy correlate inversely with 6‐min walk tests in patients with ongoing symptoms after COVID‐19.

## 1 INTRODUCTION

Recent studies have reported sustained symptoms in a significant proportion of patients following acute SARS‐CoV‐2 infection.^1^, ^2^ Patients with this “long COVID” syndrome complain of persistent breathlessness, fatigue, and decreased exercise tolerance.^2^ Although the biological mechanisms underlying these ongoing symptoms remain unknown, we recently reported that persistent increased D‐dimer levels were present in approximately 25% of convalescent COVID‐19 patients up to 4 months following the apparent resolution of their acute infection.^3^ Importantly, these increased D‐dimers were seen in a significant number of both hospitalized and nonhospitalized COVID‐19 patients, respectively. Similarly, von Meijenfeldt et al also observed persistently elevated D‐dimers in convalescent COVID‐19 patients at 4 months after hospital discharge.^4^ Moreover, sustained prothrombotic changes in thrombin‐generating capacity were also reported. Critically, however, the biological mechanisms underlying these persistent procoagulant effects following acute COVID‐19 remain poorly understood.

Postmortem studies in acute COVID‐19 have demonstrated disseminated thrombosis throughout the pulmonary vasculature.^5^, ^6^, ^7^ These thrombi are platelet‐ and fibrin‐rich, and also contain neutrophils, neutrophil extracellular traps (NETs) and activated factor XII (FXII) that triggers the contact pathway.^7^, ^8^ Current evidence suggests pulmonary thrombi in patients with severe COVID‐19 likely arise in situ within the lungs, rather than being embolic in origin.^7^, ^9^ Autopsy studies have consistently highlighted that marked pulmonary endotheliopathy is a characteristic feature of severe COVID‐19.^5^, ^7^, ^8^, ^9^ Consistent with these pathological findings, plasma markers of endothelial cell (EC) activation including von Willebrand factor antigen (VWF:Ag),^10^, ^11^, ^12^, ^13^, ^14^, ^15^ VWF propeptide (VWFpp),^16^ and soluble thrombomodulin (sTM)^10^ are all markedly elevated in patients with severe COVID‐19. Importantly, these EC biomarkers also correlate with disease severity.^10^, ^12^, ^16^, ^17^ Given the key roles of endotheliopathy and immunothrombosis in modulating the pathogenesis of acute SARS‐CoV‐2,^18^ we hypothesized that persistent EC activation might be important in modulating ongoing procoagulant effects in convalescent COVID‐19 patients and thereby contribute to the pathogenesis underpinning long COVID.

## 2 METHODS

Consecutive adult patients were enrolled from the post‐COVID‐19 review clinic in St James's Hospital, Dublin, between May and September 2020. Patients were assessed at a minimum of 6 weeks following either symptom resolution in nonhospitalized patients or hospital discharge for those requiring admission.^3^ Informed written consent was obtained from all participants and ethical approval was obtained from the St James's Hospital Research Ethics Committee. A control group of nonhospitalized asymptomatic controls (*n* = 17, mean age 47 ± 12 years) were also recruited. Plasma VWF:Ag, VWFpp, FVIII:C, and sTM were analyzed as previously described.^16^ Thrombin generation was performed in a Fluouroskan Ascent Fluorometer with Thrombinoscope software (Stago) using PPP Low reagent (1 pM tissue factor, 4 mM phospholipids) as before.^19^ Additionally, the release of extracellular DNA was measured using the fluorescent DNA‐intercalating dye Sytox Green (Invitrogen) and DNase activity was assessed by an *in vitro* NET degradation assay.^20^, ^21^ Activation of the contact factor pathway was evaluated by photometric measurement using conversion of the chromogenic substrate S‐2302 (Chromogenix).^22^ Clinical assessment at time of outpatient review included: chest x‐ray, 6‐min walk test (6MWT), measuring distance covered, lowest arterial oxygen saturation, and maximal exertion (using a Modified Borg Scale). Fatigue scores were assessed using the Chalder fatigue scale.^23^ Statistical analyses were performed using the Mann‐Whitney *U* tests and the Spearman rank correlation in GraphPad Prism 9.0 (GraphPad Software, USA) with a *p* value of < .05 considered statistically significant.

## 3 RESULTS AND DISCUSSION

Fifty convalescent COVID‐19 patients (mean age 50 ± 17 years) were assessed at a median of 68 (interquartile range 61.3–72) days following COVID‐19 symptom resolution or hospital discharge (Table S1). The majority of patients (37/50, 74%) required hospitalization during their acute COVID‐19 illness and eight patients (16%) required intensive care unit admission. Median body mass index was 28 kg/m^2^ (interquartile range 25–32) and comorbidities were apparent in 31 patients (31/50, 62%) (Table S1). All hospitalized patients received weight‐ and renally adjusted low molecular weight heparin prophylaxis during their inpatient stay. Conversely, nonhospitalized and discharged patients did not receive thromboprophylaxis and none of the cohort was on anticoagulation at time of convalescent clinic review.

**FIGURE 1:**
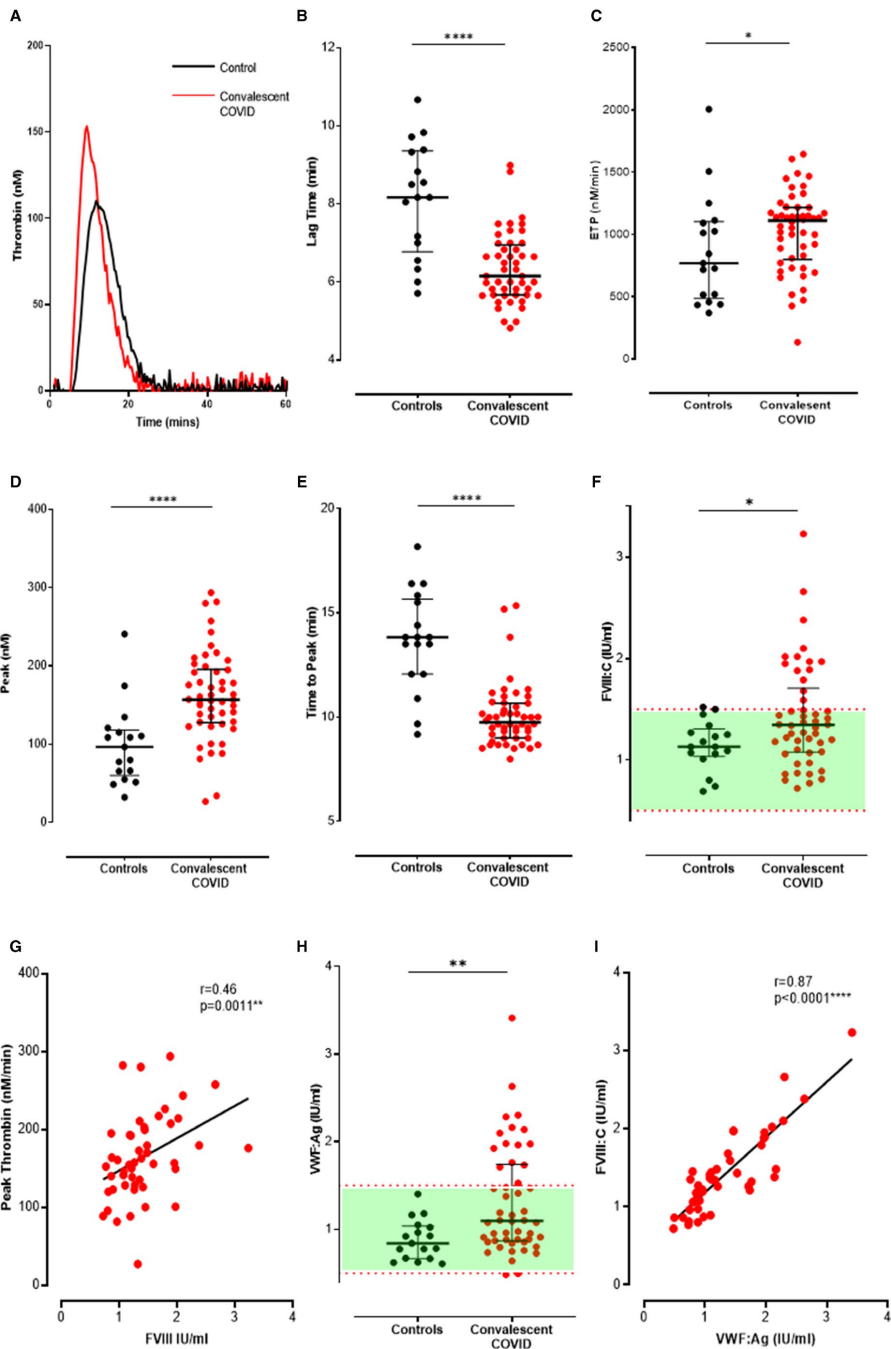
Representative thrombin generation curves (A); each curve shows the mean of duplicate reactions from one individual's plasma. The quantitative parameters of (B) lag time, (C) endogenous thrombin potential (ETP), (D) peak thrombin, and (E) time to peak were derived from the thrombin generation curves, comparing convalescent COVID‐19 (*n* = 50) and healthy controls (*n* = 17). Comparison of EC activation parameters between convalescent COVID‐19 (*n* = 50) and healthy controls (*n* = 17) are shown, including (F) FVIII:C and (H) VWF:Ag. Data are presented as median and the interquartile range. Comparisons between groups were assessed by the Mann‐Whitney *U* test. Dotted lines represent the upper and lower limits of the local normal range with results in the green shaded areas falling within the normal reference range. Correlations are shown between plasma levels of FVIII:C and (G) Peak thrombin and (I) VWF:Ag. Correlations were evaluated using the Spearman rank correlation test. (*p < .05, **p < .01, ****p < .0001). EC, endothelial cell; FVIII, factor FVIII; VWF:Ag, von Willebrand factor antigen

In keeping with the recent report of von Meijenfeldt et al,^4^ significantly enhanced thrombin generation was observed in convalescent COVID‐19 patients (Figure 1A–E and Figure S1A,B). Compared with healthy controls, lag times were significantly shorter in convalescent COVID‐19 patients (medians 6.2 min vs. 8.2 min, *p* < .0001, 95% CI −2.57 to −1.02) (Figure 1B). Additionally, endogenous thrombin potential and peak thrombin were both significantly higher in convalescent COVID‐19 patients (median endogenous thrombin potential: 1111 nM/min vs. 768 nM/min, *p* = .04, 95% CI 15–416; median peak thrombin: 157 nM vs. 97 nM, *p* < .0001, 95% CI 39–93) (Figure 1C–D). Convalescent COVID‐19 patients also demonstrated a shorter time to peak compared with controls (median 9.8 min vs. 13.8 min, *p* < .0001, 95% CI −4.8 to −2.7) (Figure 1E). To investigate whether ongoing contact pathway activation was responsible for the effects on thrombin generation, FXII activation was assessed. No significant differences in systemic FXIIa levels were seen between a subset of convalescent patients (*n* = 20) and controls (*n* = 17) (*p* = .16, 95% CI −0.15 to 0.03) (Figure S1C). Elevated plasma FVIII:C levels have previously been reported in patients with acute COVID‐19 and are known to influence thrombin generation. In the convalescent COVID‐19 patient cohort, plasma FVIII:C levels remained significantly increased compared with controls (median 1.53 IU/ml vs. 1.13 IU/ml; *p* = .04, 95% CI 0.03–0.44) with 14 (28%) patients having FVIII:C levels >1.5 IU/ml (Figure 1F). Notably, one patient with FVIII:C of 2.7 IU/ml at follow‐up 84 days postacute illness was later readmitted with pulmonary embolism. In addition, FVIII:C levels correlated with peak thrombin generation (*p* = .001) (Figure 1G). Together, these findings suggest that persistent increases in plasma FVIII levels contribute to ongoing increased thrombin generation potential in a significant proportion of convalescent COVID‐19 patients.

We next investigated the mechanism(s) responsible for the sustained elevation in plasma FVIII:C levels in convalescent COVID‐19 patients. Increased FVIII levels are associated with acute phase responses.^24^ However, in contrast to the significant elevation seen in FVIII:C levels in convalescent COVID‐19 patients, acute phase markers (including C‐reactive protein, neutrophil and white cell counts, interleukin‐6, and sCD25 levels) had normalized in most patients (Table S1 and Figure S1D,E). Although NETosis has been implicated in acute COVID‐19 immunothrombosis and endotheliopathy,^8^, ^9^ comparison of NETosis parameters in blood collected by peripheral venipuncture including DNase activity and extracellular DNA in a subset of convalescent patients and controls did not reveal any differences (median DNase activity convalescent COVID‐19 (*n* = 20): 22 792 AU vs. controls (*n* = 8): 18 835 AU; *p* = .53 95% CI −4554 to 9410 and median extracellular DNA convalescent COVID‐19 (*n* = 16): 0.37 μg/ml, vs. controls (*n* = 4): 0.37 μg/ml; *p* = .73, 95% CI −0.04 to 0.05) (Figure S1F–G).

**FIGURE 2:**
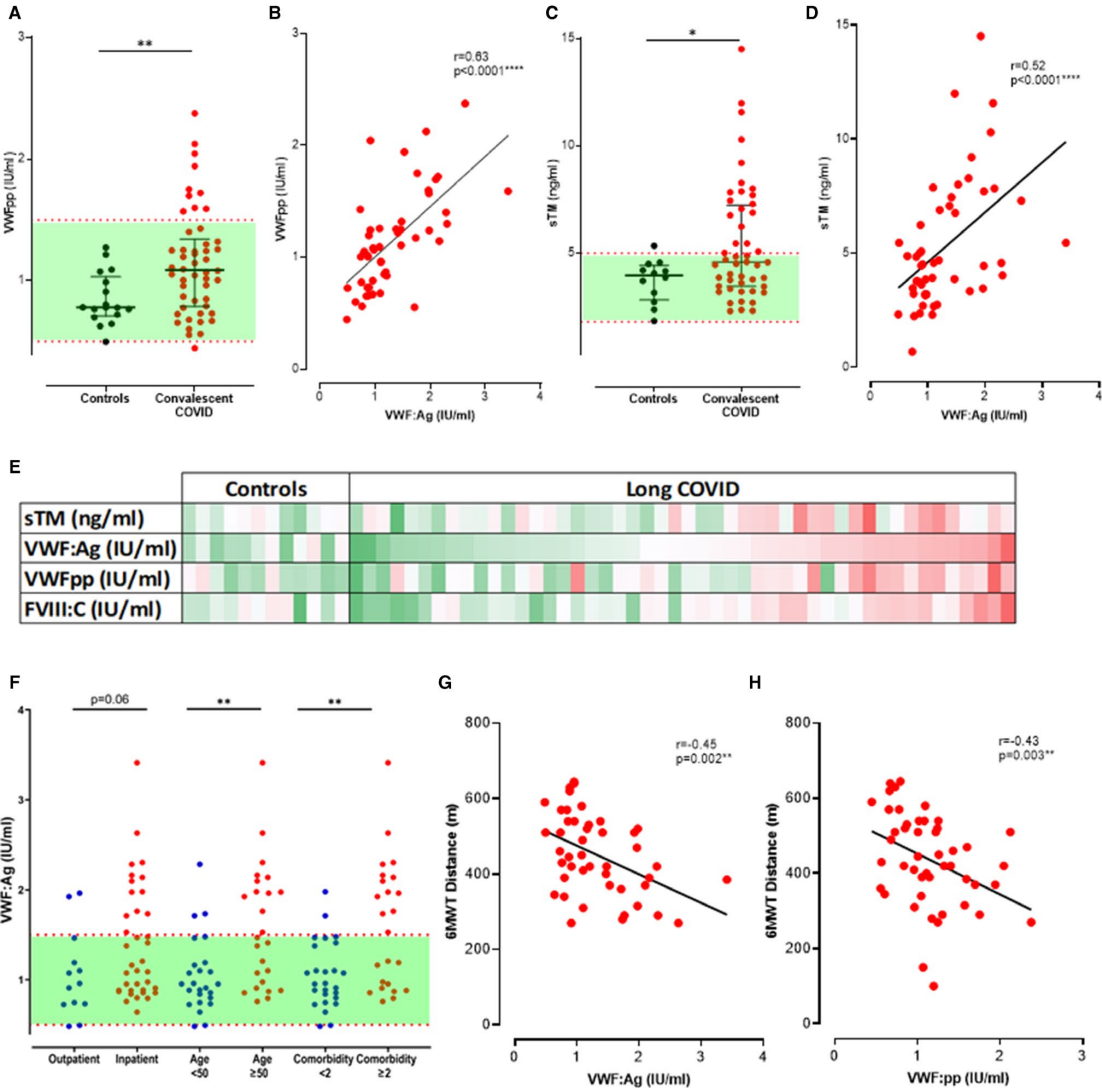
Comparison of EC activation parameters between convalescent COVID‐19 (*n* = 50) and healthy controls (*n* = 17) including: (A) VWFpp and (C) sTM. Data are presented as median and the interquartile range. Comparisons between groups were assessed by the Mann‐Whitney *U* test. Dotted lines represent the upper and lower limits of the local normal range with results in the green shaded areas falling within the normal reference range. Correlations are shown between plasma levels of (B) VWF:Ag vs. VWFpp and (D) VWF:Ag vs. sTM. (E) Heatmap visualization indicating EC marker levels detected in each subject (columns) for each protein (rows). (F) Convalescent VWF:Ag results are grouped according to whether acute infection was managed as an outpatient or inpatient; patients were aged ≥50 or <50 years and whether comorbidity counts were ≥2 or <2, respectively. Correlations are shown between 6‐min walk test distance and plasma levels of (G) VWF:Ag and (H) VWFpp, respectively. Correlations were evaluated using the Spearman rank correlation test. (ns, not significant, *p < .05, **p < .01, ****p < .0001). EC, endothelial cell; VWF:Ag, von Willebrand factor antigen; VWFpp, von Willebrand factor propeptide

In normal plasma, the majority of FVIII circulates in high‐affinity complex with VWF. Moreover, both FVIII and VWF are predominantly synthesized within EC.^25^ We observed that plasma VWF:Ag levels were also significantly increased in convalescent COVID‐19 patients compared with controls (median 1.1 IU/ml vs. 0.84 IU/ml; *p* = .004, 95% CI 0.09–0.57) (Figure 1H). Marked interindividual variation was observed, with VWF:Ag levels ranging from 0.48 to 3.4 IU/ml in convalescence. Notably, VWF:Ag levels above the upper limit of normal were observed in 15 patients (30%) with median VWF:Ag 2.0 IU/ml in this subgroup (Figure 1H). In addition, FVIII:C levels also correlated strongly with VWF:Ag levels (*r* = 0.87; *p* < .0001) (Figure 1I). During posttranslational modification within ECs, an N‐terminal 741 residue VWFpp is cleaved from each VWF monomer. VWF:Ag and VWFpp are subsequently stored together within WPB and co‐secreted in equimolar amounts following EC activation. We recently reported markedly elevated VWFpp levels in acute COVID‐19 and found that these levels correlated inversely with clinical outcome.^16^ Interestingly, VWFpp levels were also significantly elevated in convalescent COVID‐19 patients compared with controls (*p* = .009, 95% CI 0.06–0.5) (Figure 2A). Interpatient variation was again observed, with VWFpp levels being above the upper limit of normal in 10 patients (20%). Consistent with the concept of ongoing endotheliopathy, VWFpp levels also correlated strongly with VWF:Ag levels (*r* = 0.87; *p* < .0001) (Figure 2B). Collectively, these data demonstrate that persistent endotheliopathy is a common finding in convalescent COVID‐19 patients.

Thrombomodulin (TM) is an EC surface receptor that facilitates thrombin‐induced activation of protein C on EC surfaces.^26^ Recently, Giri et al reported that TM plays a key role in maintaining EC quiescence.^27^ Of particular relevance, they showed that VWF expression and secretion was markedly increased in TM‐deficient ECs. Interestingly, Goshua et al also demonstrated increased shedding of TM from EC in patients with acute COVID‐19. Furthermore, sTM levels were found to represent an independent prognostic biomarker.^10^ Given these data, we proceeded to investigate sTM levels in our cohort of convalescent COVID‐19 patients. We observed that sTM levels remained significantly elevated in convalescent COVID‐19 compared with controls (median 5.3 vs. 4.1 ng/ml; *p* = .02, 95% CI 0.01–2.7) (Figure 2C). Interestingly, the highest sTM level (14.4 ng/ml) was observed in a patient who did not require hospitalization, suggesting that sustained endotheliopathy during convalescence is not restricted to those who experienced severe COVID‐19. Consistent with the concept that loss of TM may be associated with reduced EC quiescence, we observed a significant correlation between sTM levels and plasma VWF:Ag levels (Figure 2D). Highest levels of all the EC activation parameters studied were consistently observed in the convalescent COVID cohort (Figure 2E).

To examine factors that influence sustained endotheliopathy following COVID‐19, clinical parameters including age, comorbidities, and severity of acute illness were assessed. On univariate analysis, plasma VWF:Ag, VWFpp, FVIII:C, and sTM were all significantly higher in patients who had required hospital admission during their acute COVID‐19; in patients aged ≥50 years and in patients with two or more comorbidities (Figure 2F and Figure S2A–C). These data are consistent with our previous observations that these same parameters also correlate with persistent elevated D‐dimer levels in convalescent COVID‐19 patients.^3^ Finally, we investigated the relationship between markers of sustained endotheliopathy and clinical symptoms associated with long COVID syndrome in our cohort. Interestingly, significant inverse correlations were observed on univariate analysis between 6MWT distances vs. both VWF:Ag and VWFpp, respectively (Figure 2G–H). Given this association, we sought to further evaluate using multivariable linear regression analysis including the common confounders of age, sex, and severity of initial infection. Following adjustment, however, a significant relationship between 6MWT distance and VWF:Ag (beta coefficient −4.4, 95% CI −65.5 to 56.7, *p* = .89) and VWFpp (beta coefficient −64.2, 95% CI −157.8 to 29.3, *p* = .17) was no longer seen.

Our study has some limitations. These include the small number of cases, the limited period after the acute infection, and the observational and retrospective design. In addition, the relationship between clinical outcome measures such as the 6MWT may be confounded by old age and comorbidities. Finally, EC activation is not unique to COVID‐19. EC activation and dysfunction have also been described to play important roles in the pathogenesis of other severe viral illnesses. including influenza.^28^, ^29^ However, specific differences in vascular perturbance between acute COVID‐19 and influenza have also been described. Ackermann et al showed that at autopsy, alveolar capillary microthrombi were 9 times more prevalent in patients with COVID‐19 compared with patients with influenza.^5^ Moreover, new pulmonary vessel formation was also significantly higher in COVID‐19 patients, with prominent intussusceptive angiogenesis. In addition, Stals et al recently reported that thrombotic complications were significantly higher in hospitalized patients with acute COVID‐19 compared with influenza.^30^ Collectively, these data suggest that are some similarities but also important differences vis‐a‐vis pathogenesis, endotheliopathy and immunothrombosis between acute COVID‐19 and other acute viral infections. To better understand the potential translational significance of our findings, additional studies including “omics” and imaging that directly compare EC activation and dysfunction in convalescent COVID‐19 patients as opposed to patients recovering from other types of severe viral illness will be essential.

In conclusion, our data demonstrate for the first time that sustained EC activation is common up to 10 weeks following acute SARS‐CoV‐2 infection. Importantly, this persistent endotheliopathy appears to occur independently of ongoing acute phase response or NETosis and is associated with enhanced thrombin generation potential. We postulate that shedding of TM from EC may play a role in modulating the loss of normal EC quiescence. These findings are interesting given the critical role played by endotheliopathy in the pathogenesis of acute COVID‐19. However, further adequately powered clinical trials will be required to determine whether this sustained EC activation and coagulation activation has a role in (1) stratifying patients at increased risk of thrombotic events after resolution of acute SARS‐CoV‐2 infection who may benefit from extended duration postdischarge thromboprophylaxis and/or (2) the pathogenesis of long COVID syndrome.

## CONFLICT OF INTEREST

James S. O’Donnell has served on the speaker's bureau for Baxter, Bayer, Novo Nordisk, Sobi, Boehringer Ingelheim, Leo Pharma, Takeda, and Octapharma. He has also served on the advisory boards of Baxter, Sobi, Bayer, Octapharma CSL Behring, Daiichi Sankyo, Boehringer Ingelheim, Takeda, and Pfizer and has also received research grant funding awards from 3 M, Baxter, Bayer, Pfizer, Shire, Takeda, and Novo Nordisk. The remaining authors have no conflicts of interest to declare.

## AUTHOR CONTRIBUTION

Helen Fogerty, Soracha E. Ward, Liam Townsend, Azaz Ahmad, Hannah Morrin, Claire Comerford, Cliona Ni Cheallaigh, Colm Bergin, Jamie M. O’Sullivan, and James S. O’Donnell undertook conception, patient enrollment, data collection, and interpretation. All authors contributed to literature review, final draft writing, and critical revision. All the authors have participated sufficiently in this work, take public responsibility for the content, and have made substantial contributions to this research.

## Supporting information

Fig S1

Fig S2

Tab S1

## 1

Additional investigators of the Irish COVID‐19 Vasculopathy Study (ICVS).

Niamh O’Connell (National Coagulation Centre, St James's Hospital, Dublin), Kevin Ryan (National Coagulation Centre, St James's Hospital, Dublin), Mary Byrne (National Coagulation Centre, St James's Hospital, Dublin), Dermot Kenny (Irish Centre for Vascular Biology, Royal College of Surgeons in Ireland), and Judicael Fazavana (Irish Centre for Vascular Biology, Royal College of Surgeons in Ireland).
