# Supplementary figures and images for: Persistent endotheliopathy in the pathogenesis of long COVID syndrome
Source: J Thromb Haemost. 2021 Sep 12;19(10):2546–53. doi: 10.1111/jth.15490 (PMC8420256; doi:10.1111/jth.15490)

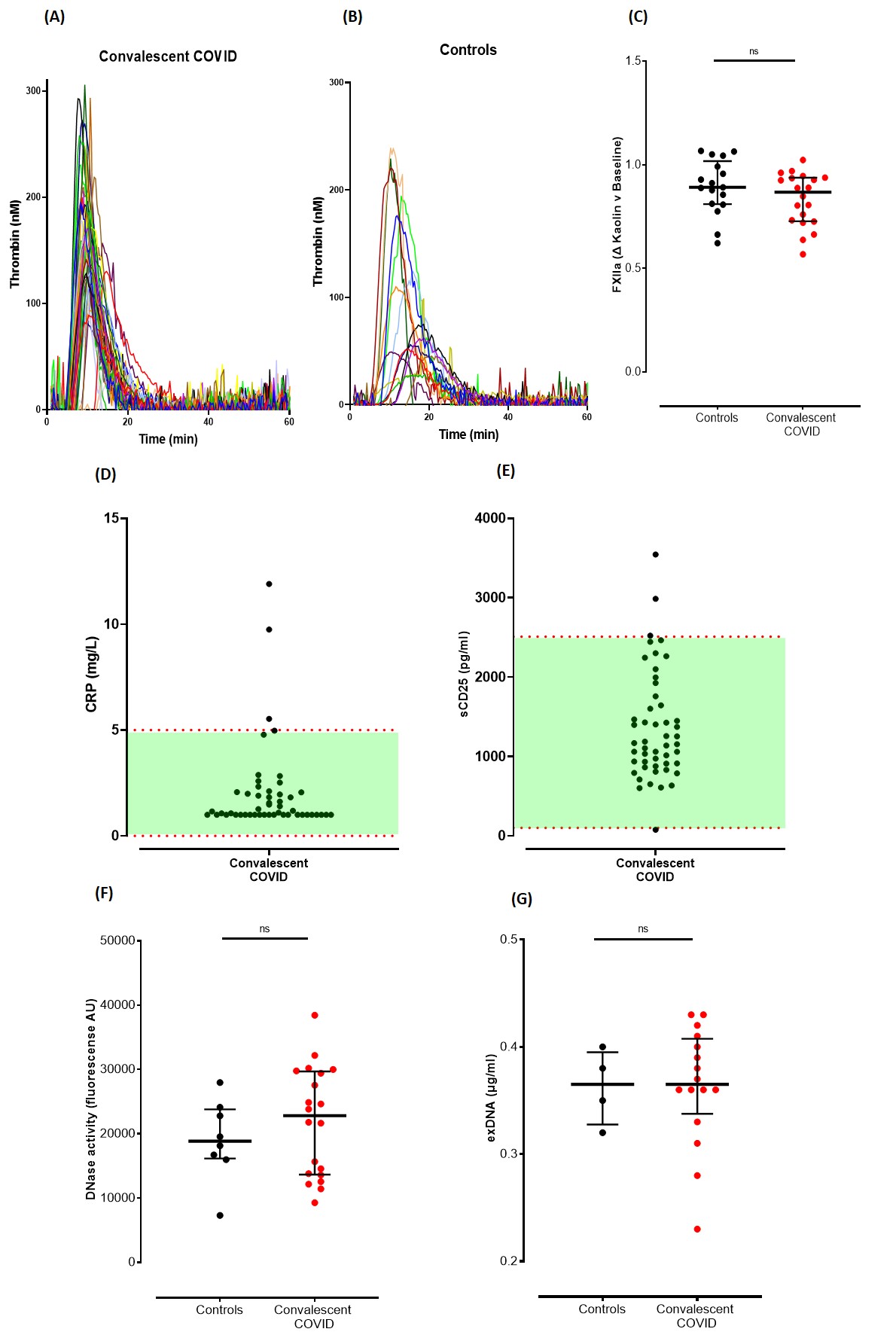

Supplement: Supplementary file 1 — Fig S1 [file JTH-19-2546-s001.jpg]

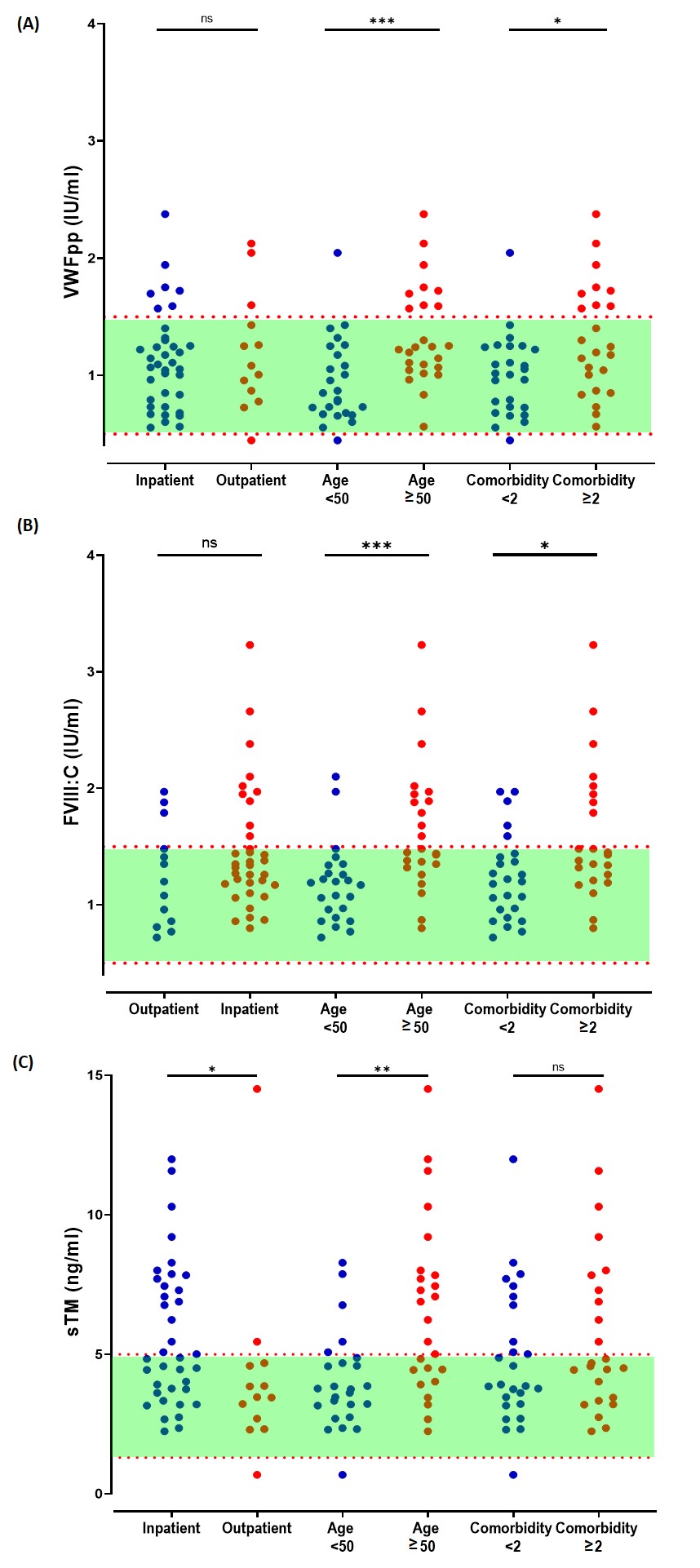

Supplement: Supplementary file 2 — Fig S2 [file JTH-19-2546-s002.jpg]
