# Supplementary material for: Persistent endotheliopathy in the pathogenesis of long COVID syndrome
Source: J Thromb Haemost. 2021 Sep 12;19(10):2546–53. doi: 10.1111/jth.15490 (PMC8420256; doi:10.1111/jth.15490)
Supplement: Supplementary file 3 — Tab S1 [file JTH-19-2546-s003.docx]

**Supplementary Table 1**

| **Parameters** | **Normal range** | **Total Cohort (n=50)** |
| --- | --- | --- |
| **Demographic data** | | |
| **Age, years (SD)** |  | 49.5 (16.5) |
| **Male – n (%)** |  | 30 (60) |
| **BMI, median (IQR)** |  | 28 (25 – 32) |
| **Comorbidity count, median (IQR)** |  | 1 (0 – 2) |
| **Hospitalization – n (%)** |  | 37 (74) |
| **ICU – n (%)** |  | 8 (16) |
| **Time to follow-up, median (IQR)** |  | 68 (61.3 – 72) |
| **Laboratory parameters – median (IQR)** | | |
| **D-dimer (ng/mL)** | 0 – 500 | 377 (243 – 495) |
| **Leukocytes (x10^9^/L)** | 4-11 | 6.3 (5.4 – 7.1) |
| **Neutrophils (x10^9^/L)** | 2-7.5 | 3.3 (4.1 – 2.9) |
| **Platelets (x10^9^/L)** | 140-450 | 272 (240 – 300) |
| **CRP (mg/mL)** | 0 – 5 | 1.1 (1 – 2) |
| **IL-6 (pg/mL)** | 0 – 7.26 | 0 (0 – 3.6) |
| **sCD25 (pg/mL)** | 101.8 – 2509.4 | 1162 (912 – 1634) |
| **Clinical parameters – median (IQR) unless otherwise stated** | | |
| **6 MWT distance (m)** | 400-700 | 430 (365 – 525) |
| **Lowest desaturation (%)** |  | 95 (94 – 96) |
| **Maximal Borg Score** |  | 3 (2 – 5) |
| **Abnormal Chest X-ray – n (%)** |  | 3 (6) |
| **Chalder Fatigue Score** |  | 14 (11 – 18) |
